# The Arabidopsis Proteins AtNHR2A and AtNHR2B Are Multi-Functional Proteins Integrating Plant Immunity With Other Biological Processes

**DOI:** 10.3389/fpls.2020.00232

**Published:** 2020-03-04

**Authors:** Raksha Singh, Rohana Liyanage, Chirag Gupta, Jackson O. Lay, Andy Pereira, Clemencia M. Rojas

**Affiliations:** ^1^Department of Plant Pathology, University of Arkansas, Fayetteville, AR, United States; ^2^Crop Production and Pest Control Research Unit, U.S. Department of Agriculture-Agricultural Research Service, Purdue University, West Lafayette, IN, United States; ^3^Department of Chemistry and Biochemistry, University of Arkansas, Fayetteville, AR, United States; ^4^Department of Crop, Soil and Environmental Sciences, University of Arkansas, Fayetteville, AR, United States

**Keywords:** nonhost resistance, co-immunoprecipitation, mass spectrometry, protein–protein interactions, interactome network

## Abstract

AtNHR2A (*Arabidopsis thaliana* nonhost resistance 2A) and AtNHR2B (*Arabidopsis thaliana* nonhost resistance 2B) are two proteins that participate in nonhost resistance, a broad-spectrum mechanism of plant immunity that protects plants against the majority of potential pathogens. AtNHR2A and AtNHR2B are localized to the cytoplasm, chloroplasts, and other subcellular compartments of unknown identity. The multiple localizations of AtNHR2A and AtNHR2B suggest that these two proteins are highly dynamic and versatile, likely participating in multiple biological processes. In spite of their importance, the specific functions of AtNHR2A and AtNHR2B have not been elucidated. Thus, to aid in the functional characterization of these two proteins and identify the biological processes in which these proteins operate, we used immunoprecipitation coupled with mass spectrometry (IP-MS) to identify proteins interacting with AtNHR2A and AtNHR2B and to generate their interactome network. Further validation of three of the identified proteins provided new insights into specific pathways and processes related to plant immunity where AtNHR2A and AtNHR2B participate. Moreover, the comprehensive analysis of the AtNHR2A- and AtNHR2B-interacting proteins using published empirical information revealed that the functions of AtNHR2A and AtNHR2B are not limited to plant immunity but encompass other biological processes.

## Introduction

Plant-pathogen interactions are determined by the genetic characteristics of the biological partners that participate in the interaction: a specific plant species, and a specific pathogen species or its biological variants. Depending on the combination of plant and pathogen, plants are classified as hosts and nonhosts. A host plant is the one that due to its genetic characteristics provides a suitable environment for pathogen growth, and as a result, succumbs to infections by such pathogen. In contrast, a nonhost plant is one that does not provide a suitable environment for pathogen growth and, consequently, is resistant to pathogen infections. This species-specific and broad-spectrum mechanism of resistance against pathogens is called nonhost resistance (Heath, 2000). Nonhost resistance is a complex process that comprises preformed and inducible defenses, as well as the participation of hundreds of genes, many of which remain to be identified and characterized (Senthil-Kumar and Mysore, 2013). Identifying all the components of nonhost resistance and understanding how it operates is of paramount importance toward generating crops with broad resistance to microbial diseases (Lee et al., 2016).

We previously identified AtNHR2A (*Arabidopsis thaliana* nonhost resistant protein 2A) and AtNHR2B (*Arabidopsis thaliana* nonhost resistance 2B) as two proteins that participate in nonhost resistance (Singh et al., 2018). We showed that these proteins are synthesized in response to infection by bacterial pathogens and contribute to the strengthening of the plant cell wall through the deposition of the ß- 1,3-glucan polymer, callose (Singh et al., 2018). *AtNHR2A* and *AtNHR2B* fusions to the green fluorescent protein (*GFP*) revealed that both proteins localized predominantly to cytoplasm, chloroplasts, and other subcellular components of unknown identity (Singh et al., 2018). Because the detailed functions of AtNHR2A and AtNHR2B are still unknown, the main objective of this work is to gain insight into their function by investigating their respective protein–protein interaction network (interactome). Investigating protein–protein interactions are powerful tools to gain insight into the function of proteins of interest given that most biological processes occur by the formation of protein complexes (Struk et al., 2018).

Since *AtNHR2A* and *AtNHR2B* are not expressed during development but induced upon exposure to pathogens or pathogen-derived elicitors (Singh et al., 2018), unraveling their respective interactomes necessitates the use of *in vivo* approaches that capture the appropriate biological context of where and when these proteins are actually present. Those approaches have mostly relied on mass spectrometry (MS), in particular affinity purification (AP) coupled with mass spectrometry (AP-MS) (Gingras et al., 2007). In an AP-MS approach, the AP step involves the purification of the protein of interest (bait) by enrichment with a ligand coupled to a solid support. The most frequently used ligands are antibodies that either directly recognize the bait or recognize an epitope tag fused to such bait. Enrichment of the bait by antibodies (immunoprecipitation), co-immunoprecipitates the interacting partners (preys) (Dunham et al., 2012). The co-immunoprecipitated samples are then proteolytically digested, and the resulting peptides are further identified by MS, hence IP-MS (Wendrich et al., 2017; Junkova et al., 2018; Zhang et al., 2019).

Here, we used IP-MS as a tool to screen for proteins that specifically interact with AtNHR2A and AtNHR2B in the biological context when *AtNHR2A* and *AtNHR2B* are optimally expressed. IP-MS of AtNHR2A-GFP and AtNHR2B-GFP revealed that AtNHR2A- and AtNHR2B-interacting proteins form complex networks spanning distinct biological processes and occurring in distinct cellular compartments. Intriguingly, in spite of the 65% amino acid sequence identity between AtNHR2A and AtNHR2B, most of the interacting proteins interacted with either AtNHR2A or AtNHR2B and only a few interacted with both proteins. The distinct interactomes of AtNHR2A and AtNHR2B highlight unique functions for each protein, and also supports our previous hypothesis that both proteins act together during plant immunity (Singh et al., 2018). Interestingly, the data revealed that in addition to interacting with proteins that participate in plant immunity, AtNHR2A and AtNHR2B also interact with proteins not implicated in plant immunity, suggesting that AtNHR2A and AtNHR2B integrate plant immunity with other biological processes.

## Materials and Methods

### Bacterial Strains

*Pseudomonas syringae* pathovar tabaci was cultured in King’s B (KB) medium at 30°C supplemented with rifampicin (25 μg/mL). *Escherichia coli* (*E. coli*) strains DH5α and Rosetta were cultured in Luria-Bertani (LB) medium at 37°C supplemented with appropriate antibiotics. *Agrobacterium tumefaciens* was cultured at 28°C in LB medium supplemented with rifampicin (25 μg/mL) and kanamycin (50 μg/mL).

### Plant Material and Growth Conditions

Arabidopsis complementation lines expressing *AtNHR2A-GFP* and *AtNHR2B-GFP* in their respective mutant backgrounds (Singh et al., 2018) and a line expressing *35Spro*:*GFP* were used for co-immunoprecipitation experiments. Seeds were surface-sterilized, plated on Murashige and Skoog (MS) media, and grown for 4 weeks at 21°C in a growth chamber with 8/16 h light/dark cycle.

*N. benthamiana* plants used for transient expression were grown in a growth chamber under 8/16 h light/dark cycle at 25°C.

### Protein Extraction From *Arabidopsis thaliana*

Four-week-old complementation lines expressing *AtNHR2A-GFP* and *AtNHR2B-GFP* in the *Atnhr2a* and *Atnhr2b* mutant backgrounds, respectively (Singh et al., 2018), and one line expressing *GFP* were flood-inoculated (Ishiga et al., 2011) with *P. syringae* pv. tabaci at 1 × 10^6^CFU/mL to induce expression of *AtNHR2A-GFP* and *AtNHR2B-GFP*. Inoculated leaves were collected at 6 hpi and flash frozen in liquid nitrogen. Approximately 1 g of tissue (from 20 plants/genotype/treatment) was manually ground and homogenized in 6 mL of Co-IP extraction buffer [100 mM Tris-HCl, pH 7.5, 150 mM NaCl, 1 mM EDTA, 10 mM MgCl_2_, 10% Glycerol, 0.2% Nonidet P-40, 1 mM phenylmethylsulfonyl fluoride (PMSF), 5 mM dithiothreitol (DTT), and 1X Proteinase inhibitor cocktail (Sigma Aldrich, St. Louis, MO, United States)]. Extracts were incubated in ice for 30 min and centrifuged twice at 13,000 rpm for 30 min at 4°C. Clear supernatants were transferred to new pre-chilled 50-mL falcon tubes and total protein concentrations were measured using Bradford Assay (BioRad, Hercules, CA, United States). Protein expression was confirmed by Western blot using anti-GFP-HRP (1:1000 dilution; Miltenyi Biotec, Auburn, CA, United States) and detected by luminol solution (ImmunoCruz, SantaCruz Biotechnology Inc., Dallas, TX, United States) (Supplementary Figure S1A).

### Co-immunoprecipitation (Co-IP)

**FIGURE 1:**
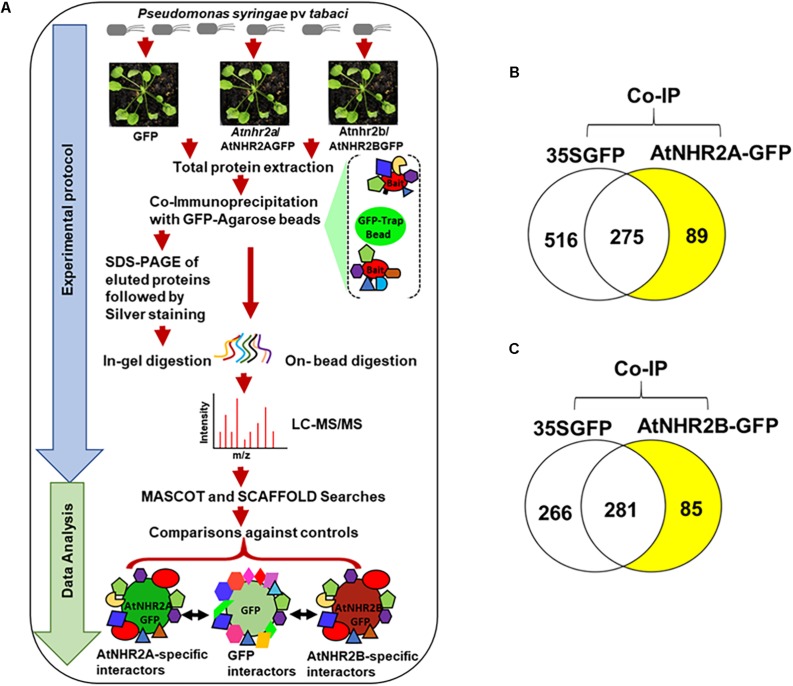
Co-immunoprecipitation/mass spectrometry analysis to unravel the AtNHR2A and AtNHR2B Interactomes. Co-IP/MS workflow summarizing experimental protocol and data analysis. For the experimental protocol, four-week-old transgenic *Arabidopsis thaliana* plants expressing AtNHR2A-GFP and AtNHR2B-GFP GFP were inoculated with *Pseudomonas syringae* pv tabaci at 1 × 10^6^ CFU/mL. At 6 hpi, plants were harvested for total protein extraction. Extracted proteins from each sample were subjected to co-immunoprecipitation using GFP Trap A beads. GFP-bound protein complexes were eluted with 2X SDS sample buffer and resolved by SDS-PAGE for silver staining and in-gel trypsin digestion, or directly processed for on-bead trypsin digestion. Both in-gel and on-bead tryptic digested products were analyzed by LC-MS/MS. For data analysis, identified peptide spectra were then analyzed by Mascot using the *Arabidopsis thaliana* protein database as reference, and identified peptides were further validated and compared using Scaffold to identify peptides representing proteins that specifically interact with AtNHR2A-GFP or AtNHR2B-GFP but not with GFP. Peptide identification were done by setting peptide hits ≥ 2 with the false discovery rate (FDR) below 1% based on decoy database **(A)**. Venn diagrams show specific proteins interacting with AtNHR2A-GFP **(B)** and AtNHR2B-GFP **(C)** (shaded in yellow).

Five milligrams of total protein extracts (in 5 ml) were mixed with 200 μl of GFP-Trap A beads (ChromoTek, Germany) and incubated overnight at 4°C with end to end rocking. After incubation, beads were washed once with TEN buffer (10 mM Tris-HCl, pH 7.5, 150 mM NaCl, 0.5 mM EDTA) followed by two washes with higher stringency TEN buffer (10 mM Tris-HCl, pH 7.5, 500 mM NaCl, 0.5 mM EDTA). Proteins bound to the beads were resuspended in 2 ml of TEN buffer (10 mM Tris-HCl, pH 7.5, 150 mM NaCl, 0.5 mM EDTA). We conducted two independent co-immunoprecipitation experiments for AtNHR2A (AtNHR2A interactors 1st screen, AtNHR2A interactors 2nd screen) (Supplementary Table S1), and two independent co-immunoprecipitation experiments for AtNHR2B (AtNHR2B interactors 1st screen, AtNHR2B interactors 2nd screen) (Supplementary Table S2). For each co-immunoprecipitation experiment that included AtNHR2A-GFP, a parallel co-immunoprecipitation was done for GFP. Similarly, for each co-immunoprecipitation experiment that included AtNHR2B-GFP, a parallel co-immunoprecipitation was done for GFP. Co-immunoprecipitated samples were processed for on-bead trypsin digestion or, in-gel trypsin digestion (Figure 1A).

We used two digestion methods to increase the recovery of proteins as each method provides specific advantages. The advantage of the on-bead digestion method is that it maximizes protein solubilization making them more susceptible to enzymatic cleavage (Chen et al., 2007). The advantage of in-gel digestion method is that it increases the number of protein identifications because it reduces the complexity of the sample by adding the size separation on the gel prior to MS (Feist and Hummon, 2015). The digestion strategy also determines the instrument used for mass spectrometry.

### On-Bead Trypsin Digestion

Proteins bound to the beads were pre-digested with 0.2% of RapiGest SF (Waters Corporation, Milford, MA, United States) in 50 mM ammonium bicarbonate (Schechter et al., 2014). After pre-digestion, samples were reduced with 10 mM Tris (2-carboxyethyl) phosphine hydrochloride (TCEP-HCl) and alkylated with 25 mM iodoacetamide, followed by overnight digestion at 37°C with sequencing grade trypsin (Promega, Madison, WI, United States) in 1:50 trypsin/protein ratio. Digested samples were dissolved in 1% acetonitrile/0.1% formic acid.

### In-Gel Trypsin Digestion

Proteins bound to the beads were eluted with 100 μl of 2X SDS-buffer and boiled at 90°C for 8 min. Proteins co- immunoprecipitated with AtNHR2A-GFP, AtNHR2B-GFP, and GFP were separated by mass spectrometry compatible 4–20% Tris-Glycine precast gel (Thermo Fisher Scientific Inc., Carlsbad, CA, United States) then stained with Pierce^TM^ silver stain kit for Mass Spectrometry (Thermo Fisher Scientific Inc., Carlsbad, CA, United States). The stained gel lanes were then divided into four sections based on 4 ranges of molecular weight (Supplementary Figure S1B), and each section was sliced further into about 4–5 pieces of ca 1 mm^2^ size gel pieces to increase the trypsin contact surface area for efficient trypsin digestion. Gel pieces were first destained with 15 mM potassium ferricyanide and 50 mM sodium thiosulfate. Destained gel pieces were dehydrated using 100% HPLC grade acetonitrile and completely dried using a SpeedVac. Dried gel pieces were treated with 10 mM DTT in 25 mM ammonium bicarbonate (pH 7.8) at 60°C for 1 h to reduce proteins. Excess DTT was removed by pipetting, and gel pieces were treated with 20 mM iodoacetamide in 25 mM ammonium bicarbonate at room temperature for 1 h in the dark to alkylate the reduced proteins. After the iodoacetamide treatment, gel pieces were washed thoroughly with 25 mM ammonium bicarbonate, dehydrated with 100% acetonitrile, and completely dried using a SpeedVac. One hundred microliters of MS grade Trypsin (Thermo Fisher Scientific Inc., Carlsbad, CA, United States) in 25 mM ammonium bicarbonate were added to the dried gel pieces to a final concentration of 50 ng/μl. Samples were kept at 4°C for 30 min to ensure trypsin was efficiently absorbed to the dried gel pieces. One hundred microliters of additional 25 mM ammonium bicarbonate were added to each sample and incubated at 37°C for 24 h. Trypsin-digested samples were acidified with 5% formic acid (FA) in 60% acetonitrile (ACN) to quench trypsin enzyme activity and break down ammonium bicarbonate. The samples were further desalted using Pierce C18- spin columns (Thermo Fisher Scientific Inc., Carlsbad, CA, United States) following manufacturer’s instructions.

### Mass Spectrometry and Data Analysis

Five microliters of the on-bead protein digested samples were used for LC-MS/MS analysis using an LTQ-Orbitrap Velos Pro (Thermo Fisher Scientific, San Jose, CA, United States) coupled with a U3000 RSLCnano HPLC (Thermo Fisher Scientific, San Jose, CA, United States) using previously described conditions (Alvarez et al., 2013, 2014; Huang et al., 2016). The LTQ-Orbitrap mass analyzer was operated in the data-dependent acquisition mode in which each MS1 scan was followed by collision-induced dissociation (CID) of the 10 most intense precursor ions. The mass range for the MS1 done using the FTMS was 365 to 1800 m/z with resolving power set to 60,000 @ 400 m/z and the automatic gain control (AGC) target set to 1,000,000 ions with a maximum fill time of 100 ms. The selected precursors were fragmented in the ion trap using an isolation window of 1.5 m/z, an AGC target value of 10,000 ions, a maximum fill time of 100 ms, a normalized collision energy of 35, and activation time of 30 ms. Dynamic exclusion was performed with a repeat count of 1, exclusion duration of 45 s, and a minimum MS ion count for triggering MS/MS set to 5000 counts.

Five microliters of the in-gel desalted trypsin-digested peptides were analyzed by LC-MS/MS using an Agilent 1200 series microflow high-performance liquid chromatography (HPLC) coupled to a Bruker amaZon SL quadrupole ion trap mass spectrometer with a captive spray ionization source. Peptides were separated by reverse-phase high-performance liquid chromatography (RP-HPLC) using a Zorbax SB C18 column (150 × 0.3 mm, 3.5 μm particle size, 300Å pore size, Agilent Technologies) with a solvent flow rate of 4 μL/minute and a gradient of 5–38% consisting of 0.1% FA (solvent A) and ACN (solvent B) over a period of 320 min. MS analyses were performed in a positive ion mode using Bruker captive electrospray source with a dry nitrogen gas temperature of 200°C and a nitrogen flow rate of 3 L/minute. LC-MS/MS data were carried out in the Auto MS(n) mode. Optimized trapping condition for the ions at m/z 1000 was set. Regular MS level scans were performed using the enhanced scanning mode (8100 m/z/second), while MS/MS fragmentation (collision induced fragmentation) scans were performed automatically for top ten precursor ions with a set threshold for 1 min using UltraScan mode (32,500 m/z/second) (Karash et al., 2017; Rath et al., 2019). Immunoprecipitated samples (AtNHR2A 2nd screen and AtNHR2B 2nd screen) were injected twice into the latter instrument to generate a technical replicate (AtNHR2A 3rd screen and AtNHR2B 3rd screen) (Supplementary Tables S1, S2). Consolidated data and common interactors between AtNHR2A and AtNHR2B is presented in Supplementary Table S3.

The MS/MS spectra obtained from both methods were searched against the TAIR10_20101214 non-redundant *Arabidopsis* protein available in the Arabidopsis Information Resource (TAIR) database, containing 35,639 protein entries. MASCOT v 2.5 (Matrix Science, London, United Kingdom; version 2.5.1) database search software was used to search TAIR database using trypsin as the enzyme with one miss cleavage, carbamidomethyl fixed modification, and methionine oxidation as the variable modification. Protein identifications were accepted if they were represented by at least 2 unique peptides, each peptide showing probability higher than 80% (Mascot Ion Score higher than 20) and with a false discovery rate of < 1.0% as determined by the Scaffold Local FDR algorithm. Protein probabilities were assigned by the Protein Prophet algorithm (Keller et al., 2002). For each of the AtNHR2A screens, AtNHR2A interactors were those proteins identified only from AtNHR2A-GFP samples but not from GFP samples. Similarly, for each of the AtNHR2B screens, AtNHR2B interactors were those proteins identified only from the AtNHR2B-GFP samples and not from GFP samples. Proteins identified in all samples (AtNHR2A-GFP, AtNHR2B-GFP, and GFP) were considered non-AtNHR2A- or non-AtNHR2B- specific proteins and therefore, were removed from the list.

### Protein Annotations

AtNHR2A and AtNHR2B interacting proteins were broadly classified into biological process, molecular function, and cellular components, according to the Gene Ontology (GO) annotation tool of the *Arabidopsis thaliana* reference genome using GO Slim available in the Arabidopsis Information Resource (TAIR) database^1^. A more detailed annotation was obtained by identifying convincing empirical data regarding protein subcellular localization and function in the published literature.

### Plasmid Constructs

*AtENGD-1* (At1g30580) was cloned into *pDONR207* (Thermo Fisher Scientific Inc., Carlsbad, CA, United States) using primers 5′GGGGACAAGTTTGTACAAAAAAGCAGGCTYYA TGCCTCCGAAAGCCAAA3′ and 5′GGGGACCACTTTGTA CAAGAAAGCTGGGTYTCATTTCTTCCCACCACCG3′, and subsequently transferred to *pET59-DEST*^TM^ (EMD Millipore, Burlington, VA, United States) to generate *6X-His-AtENGD-1*.

A plasmid harboring *AtRPN1A* (At2g20580) epitope tagged with *Myc* (*Myc-AtRPN1A*) was obtained from the Arabidopsis Biological Resource Center (ABRC) stock collection^2^.

*AtCCoAOMT1* (At4g34050) was cloned into *pDONR201* (Thermo Fisher Scientific Inc., Carlsbad, CA, United States) using primers 5′GGGGACAAGTTTGTACAAAAAAGCAGGC TYYATGGCGACGACAACAACA3′ and 5′GGGGACAAGTTT GTACAAAAAAGCAGGCTYYATGCCTCCGAAAGCCAAA3′ and subsequently transferred to *pEarleyGate201* (Earley et al., 2006) to generate *HA-AtCCoAOMT1*.

Full length *AtNHR2B* and truncated *AtNHR2B_1__–__140_* in *pDONR201* were transferred to *pSITEnEYFPC1* (Martin et al., 2009). *AtCCoAOMT1* was cloned into pSITEcEYF PC1(Martin et al., 2009).

Plasmids harboring *Myc-AtRPN1A, HA-AtCCoAOMT1, AtCCoAOMT1-cEYFP, AtNHR2B-nEYFP*, and *AtNHR2B_(__1__–__140__)_-nEYFP* were transformed into *Agrobacterium tumefaciens* strain GV2260 by electroporation for transient gene expression in *N. benthamiana*. *pET59-DEST:AtENGD-1* was transformed into *E. coli* strain Rosetta for protein expression and purification.

### Protein Purification in *E. coli*

*Escherichia coli* harboring plasmid *pET59-DEST:AtENGD-1* was grown in 5 mL of LB broth supplemented with ampicillin (50 μg/mL). The overnight culture was then subcultured into 250 mL of LB supplemented with ampicillin (50 μg/mL). When the culture reached an OD_600_ of 0.5, protein expression was induced by adding 0.5 mM isopropyl-1-thio-β-d-galactopyranoside (IPTG) followed by incubation for additional 6 h at 28°C. Bacterial cells were harvested by centrifugation at 12,000 rpm at 4°C, and proteins were purified on nickel-nitrilotriacetic acid (Ni-NTA) agarose resin (Thermo Fisher Scientific Inc., Carlsbad, CA, United States), following manufacturer’s instructions. Proteins were then separated in a 12% SDS-polyacrylamide gel and stained with Coomassie Brilliant Blue to confirm IPTG induction. Protein expression was confirmed by Western blotting using anti-His antibodies (1:500 dilution; Cell Signaling Technology, Danvers, MA, United States).

### Visualization of the Integrated Interactome

The predicted functional association network of *Arabidopsis thaliana* was downloaded from the STRING database available at: https://stringdb-static.org/download/protein.links.v11.0/3702.protein.links.v11.0.txt.gz. All evidence channels (genomic context, coexpression, text mining, experimental evidence, curated protein databases) (Szklarczyk et al., 2019) were used for the analysis. The downloaded network had ∼11 million total edges with scores ranging from 150 to 900. The edges were sorted based on the increasing order of their edge score with the top 10% edges (combined minimum score of 626) selected as high confidence edges and the rest filtered. This top 10% network was searched with the 154 proteins that we found to be interacting with either AtNHR2A or AtNHR2B or with both proteins. The predicted links in STRING network and the interactions identified by IP-MS were assembled using Cytoscape v3.3.0 and including subcellular localization and functional category based on literature searches (Supplementary Tables S4–S6).

### Validation of Protein–Protein Interactions by Transient Expression in *N. benthamiana* and Co-immunoprecipitation

*Agrobacterium tumefaciens* strains harboring *HA-AtCCoAOMT1, Myc-AtRPN1A*, and *AtNHR2B-GFP* were grown overnight at 28°C with constant shaking. Overnight cultures were harvested by centrifugation at 6,000 rpm for 10 min, resuspended in induction buffer [20mM 2-N-morpholino ethanesulfonic acid (MES) pH 5.5; 3% sucrose, 200μM acetosyringone], and incubated at room temperature with constant shaking for 4 h. Induced cultures were adjusted to an OD_600_ = 0.3. *A. tumefaciens* harboring *AtNHR2B-GFP* was co-infiltrated with *A. tumefaciens* harboring *HA-AtCCoAOMT1* or *Myc-AtRPN1A* into fully expanded leaves of 3-week-old *N. benthamiana* plants using a needless syringe. *A. tumefaciens* harboring a construct encoding *GFP* was co-infiltrated with either *HA-AtCCoAOMT1* or *Myc-AtRPN1A* and used as negative control. After 3 days, infiltrated leaves were harvested for protein extraction. Proteins isolated from leaves co-expressing *AtNHR2B-GFP/HA-AtCcoAOMT* and *GFP/HA-AtCcoAOMT* were immunoprecipitated with the Pierce^TM^ HA Epitope Tag Antibody conjugated to agarose beads (Thermo Fisher Scientific Inc., Carlsbad, CA, United States). Proteins isolated from leaves co-expressing *AtNHR2B-GFP/Myc-AtRPN1A* and *GFP/Myc-AtRPN1A* were precleared with protein A-sepharose beads (GE Healthcare, Chicago, IL, United States) at 4°C for 2 h with gentle shaking. Precleared samples were immunoprecipitated with Myc antibodies (Miltenyi Biotec, Bergisch Gladbach, Germany) coupled with protein A-sepharose beads. Co-immunoprecipitated samples were washed and eluted in 2× SDS protein loading buffer, ran in a SDS-PAGE gel, and transferred to nitrocellulose membranes for Western blot and probed with anti-GFP-HRP (1:1000 dilution; Miltenyi Biotec, Bergisch Gladbach, Germany), or anti-Myc-HRP (1:1000 dilution; Miltenyi Biotec, Bergisch Gladbach, Germany), or anti-HA-HRP (1:1000 dilution; Thermo Fisher Scientific Inc., Carlsbad, CA, United States), depending on the protein combination. The proteins were detected by chemiluminescence (SantaCruz Biotechnology, Dallas, TX, United States).

### Validation of Protein–Protein Interactions by Semi-*in vivo* Co-immunoprecipitation

To validate the interaction between AtNHR2A-GFP and His-AtENGD-1, *AtNHR2A-GFP* and *GFP* were transiently expressed in *N. benthamiana*, and proteins extracted as described above. *His-AtENGD-1* was expressed and purified in *E. coli*. Thereafter, 5 μg of the purified His-AtENGD-1 were mixed with 100 μg of total protein extracted from *N. benthamiana* plants transiently expressing *AtNHR2A-GFP* or *GFP*, and subjected to co-immunoprecipitation with GFP Trap-A beads (Chromotek, Germany), as described above. The co-immunoprecipitated samples were washed, eluted in 2 × SDS protein loading buffer, resolved by SDS-PAGE, and transferred to a nitrocellulose membrane for Western blotting using anti-His (1:500 dilution; Cell Signaling Technology) or anti-GFP-HRP (1:1000 dilution; Miltenyi Biotec, Bergisch Gladbach, Germany) antibodies.

### Bimolecular Fluorescence Complementation

*AtNHR2B* fused to the N-terminal-encoding half of the enhanced yellow fluorescent protein (*EYFP*), and *AtCCoAOMT1* fused to the C-terminal-encoding half of *EYFP* were co-infiltrated in *N. benthamiana* for transient expression. Leaf samples were collected at 3 days after infiltration for live-cell image analysis using a Leica TCS SP5 II confocal microscope (Leica Microsystems, Buffalo Grove, IL, United States) at an excitation wavelength of 514 nm (argon laser) and an emission wavelength of 500 to 530 nm.

## Results

### AtNHR2A and AtNHR2B Interact With Distinct Set of Proteins Localized to Several Subcellular Compartments and Involved in Multiple Biological Processes

To identify the AtNHR2A- and AtNHR2B- interactomes, this work relied on IP-MS. The experiments used transgenic Arabidopsis lines expressing *AtNHR2A-GFP* and *AtNHR2B-GFP* in the *Atnhr2a* and *Atnhr2b* mutant backgrounds, respectively (Singh et al., 2018). To provide the appropriate biological context, we inoculated plants with *P. syringae* pv. tabaci to induce expression of *AtNHR2A* and *AtNHR2B* (Figure 1A). Because the maximum induction of both *AtNHR2A* and *AtNHR2B* occurs at 6 hours post inoculation (hpi) (Singh et al., 2018), we chose this time point to harvest inoculated plants for protein extraction. Thus, we are confident that these conditions ensured endogenous levels of AtNHR2A-GFP and AtNHR2B-GFP and reduced the occurrence of spurious results. The combination of the two approaches increased protein recovery, while the stringent data analyses allowed us to confidently identify true interactors for AtNHR2A and AtNHR2B.

The experiments using AtNHR2A-GFP as bait yielded a total of 881 nonredundant proteins from both methods. From those 881 proteins, 516 proteins interacted with the GFP tag, 275 proteins interacted with both the GFP tag and AtNHR2A-GFP, and 89 interacted only with AtNHR2A-GFP (Figure 1B and Supplementary Table S1). Similarly, co-immunoprecipitation experiments using AtNHR2B-GFP yielded 632 nonredundant proteins from both methods. Out of those 632 proteins, 266 interacted with the GFP tag, 281 interacted with both the GFP tag and AtNHR2B-GFP, and 85 interacted only with AtNHR2B-GFP (Figure 1C and Supplementary Table S2). Out of the 89 proteins interacting with AtNHR2A and the 85 proteins interacting with AtNHR2B, we found 20 proteins that interacted with both AtNHR2A and AtNHR2B (Supplementary Table S3). The AtNHR2A- and AtNHR2B- specific interactors were considered high confidence interactors because: (i) they were not identified in the negative control, (ii) were represented by two or more unique peptides, and (iii) they had an FDR less than 1.0%.

We initially classified the proteins interacting with AtNHR2A and AtNHR2B using the GO categories biological process, molecular function, and cellular component, and found that AtNHR2A- and AtNHR2B- interacting proteins were placed in similar functional categories (Supplementary Figures S2A,B), yet visual inspection indicated striking differences between the two datasets. Therefore, to extract more meaningful information from the data, we manually annotated the proteins using published literature that convincingly reported subcellular localization and/or specific function. That specific function was then assigned to a broader functional category. Only proteins with known subcellular localization and function were included (Tables 1–3).

**TABLE 1:** AtNHR2A specific interactors with known subcellular localization and function, ordered by gene ID.

**Gene ID**	**Description**	**Subcellular location**	**Function**	**References**
At1g01560	Mitogen-activated protein kinase, MAPK (AtMPK11)	Nucleus, Cytoplasm	Regulation of Plant immunity	Carrasco et al., 2014; Eschen-Lippold et al., 2016
At1g03760	Prefoldin subunit	Nucleus, cytoplasm	Protein folding	Millan-Zambrano and Chavez, 2014
At1g07650	LRR-type receptor protein kinase (BSR650)	Plasma membrane	Brassinosteroid Signaling	Xu et al., 2014
At1g09590	L21-type protein of large ribosomal subunit (AtRPL21A)	Cytoplasm	Protein synthesis	Carroll, 2013
At1g12220	R-type immune receptor (AtRPS5)	Plasma membrane	Plant immunity	Qi et al., 2012
At1g21400	2-oxoisovalerate dehydrogenase subunit α1	Mitochondria	Branched chain amino acid catabolism	Fujiki et al., 2001
At1g30580	GTPase (AtENGD-1)	Cytoplasm	Negative regulator of plant immunity	Cheung et al., 2010
At1g42950	40S ribosomal protein S7	Cytoplasm	Protein synthesis	Carroll, 2013
At1g52360	β subunit of coatomer (COP) adaptor complex	Golgi apparatus	Vesicle trafficking	Brandizzi and Barlowe, 2013
At1g60950	Ferredoxin (AtFd2)	Chloroplast	Photosynthetic electron transport	Hanke and Mulo, 2013
At1g65980	Peroxiredoxin type 2 (AtPrxIIB)	Cytoplasm	Reactive oxygen species homeostasis	Horling et al., 2002
At1g67090	Ribulose bisphosphate carboxylase small chain (RBCS1A)	Chloroplast	Carbon fixation	Vitlin Gruber and Feiz, 2018
At1g74350	Type-II-maturase-like intron splicing factor (AtnMat4)	Mitochondria, Chloroplast	RNA processing and maturation	Keren et al., 2009; Cohen et al., 2014
At1g74350	Type-II-maturase-like intron splicing factor (AtnMat4)	Mitochondria, Chloroplast	RNA processing and maturation	Keren et al., 2009; Cohen et al., 2014
At1g78300	AtGRF2/AtGF14-omega (14-3-3 family)	Mitochondria, Cytoplasm and Nucleus	Hormone signaling	Yoon and Kieber, 2013
At1g78860	Mannose-binding lectin protein (AtGAL2)	Extracellular space	Plant immunity	Hwang and Hwang, 2011; Ghahremani et al., 2018
At2g10940	Proline-rich glycoprotein (AtPRP15)	Extracellular space	Post-translational modification of cell wall proteins	Durufle et al., 2017
At2g14830	AtIST1- like10	Endosomes	Vesicle trafficking	Buono et al., 2016
At2g18020	L8-type protein of large ribosomal subunit (AtRPL8A)	Cytoplasm	Protein synthesis	Carroll, 2013
At2g18450	Succinate dehydrogenase 1-2 (AtSDH1-2)	Mitochondria	Carbohydrate metabolism	Figueroa et al., 2002
At2g27100	SERRATE (AtSE)	Nucleus	Alternative splicing	Raczynska et al., 2014
At2g43030	L3-type protein of large ribosomal subunit	Chloroplast	Protein synthesis	Kleffmann et al., 2004
At2g43610	Class-IV chitinase-like protein	Extracellular space	Plant immunity	Kumar et al., 2018
At3g11430	Glycerol-3-phosphate acyltransferase (AtGPAT5)	Endoplasmic reticulum	Glycerolipid synthesis	Chen et al., 2011
At3g19140	Day neutral flowering (AtDNF)	Plasma membrane	E3 ubiquitin ligase	Morris et al., 2010
At3g49010	60 S L13-type protein of large ribosomal subunit (AtRPL13B)	Cytoplasm	Protein synthesis	Carroll, 2013
At3g53110	Putative DEAD-box RNA helicase (AtRH38/AtLOS4)	Nucleus, cytoplasm	RNA metabolism	Gong et al., 2002
At3g54590	Extensin-type glycoprotein (AtEXT2)	Extracellular space	Cell wall biosynthesis	Liepman et al., 2010
At3g56840	L2-hydroxyglutarate dehydrogenase (L2-HGDH)	Mitochondria	Mitochondrial metabolic repair	Hudig et al., 2015
At4g02840	Nuclear Ribonucleoprotein SmD1b	Nucleus	Splicing and RNA quality control	Elvira-Matelot et al., 2016
At4g15900	Core component of spliceosome-associated MAC complex (AtPRL1/AtMAC2)	Nucleus	Regulation of plant immunity	Palma et al., 2007
At4g31570	Kinesin-related protein	Cytoplasm	Hormone homeostasis	Molesini et al., 2012
At4g36020	CSD-type RNA chaperone (AtCSP1)	Cytoplasm, Nucleus	RNA chaperone	Kim et al., 2007; Yang and Karlson, 2013
At5g02040	Prenylated RAB acceptor 1.A1 (AtPRA1.A1)	Endoplasmic reticulum	Vesicle trafficking	Alvim Kamei et al., 2008
At5g06740	Legume-lectin-type receptor-like protein kinase (AtLecRK-S.5)	Plasma membrane	Plant Immunity	Lannoo and Van Damme, 2014
At5g15700	Dual-targeted DNA-dependent RNA polymerase (AtRpoT2)	Mitochondria, Chloroplast	Transcription	Hedtke et al., 2000
At5g16400	F-type thioredoxin (AtTRX-F2)	Chloroplast	Redox regulation	Cain et al., 2009
At5g17640	Abiotic stress gene 1 (ASG1)	Plasma membrane	Abiotic stress responses	Batelli et al., 2012
At5g26000	Thioglucoside glucohydrolase (AtBGLU38/AtTGG1)	Cytoplasm	Indole glucosinolate biosynthesis	Zhao et al., 2015
At5g35740	Endo-1,3-beta-glucosidase	Extracellular space	Cell wall biosynthesis	Houston et al., 2016
At5g38420	Small subunit of ribulose bisphosphate carboxylase (AtRBCS-2B/AtS2B)	Chloroplast	Carbon fixation	Vitlin Gruber and Feiz, 2018
At5g48140	Polygalacturonase	Extracellular space	Plant immunity	Cao, 2012
At5g61780	Ribonuclease TUDOR 2 (AtTSN2)	Cytoplasm	mRNA processing	Frei dit Frey et al., 2010
ATCGOO470	ATPase ε subunit	Chloroplast	ATP synthesis	Friso et al., 2004
ATCGOOO20	Photosystem II protein D1 (PSBA)	Chloroplast	ATP synthesis	Friso et al., 2004
ATCG00780	50S Ribosomal protein L14 (RPL14)	Chloroplast	Protein synthesis	Kleffmann et al., 2004
ATMG01190	ATP synthase subunit α (ATPA)	Mitochondria	ATP synthesis	Kruft et al., 2001

**TABLE 2:** AtNHR2B specific interactors with known subcellular localization and function, ordered by gene ID.

**Gene ID**	**Description**	**Subcellular location**	**Function**	**References**
At1g02780	60S ribosomal protein L19-type (AtRPL19A)	Cytoplasm	Protein synthesis	Carroll, 2013
At1g07770	40S ribosomal protein S15a-1 (AtRPS15aA)	Cytoplasm	Protein synthesis	Carroll, 2013
At1g20010	β-tubulin (AtTUB5)	Plasma membrane	Component of cytoskeleton	Minami et al., 2009
At1g22700	Protein Pale Yellow Green7 (AtPYG7)	Chloroplast	Photosystem biogenesis	Yang H. et al., 2017
At1g41880	60S ribosomal protein L35a-type (AtRPL35aB)	Cytoplasm	Protein synthesis	Carroll, 2013
At1g45000	AtRPT4b	Nucleus/Cytoplasm	Proteasome component	Fu et al., 1999
At1g49970	CLP protease proteolytic subunit 1 (AtClpR1)	Chloroplast	Chloroplast development and differentiation	Koussevitzky et al., 2007
At1g72930	TNL-type NLR pathogen effector recognition protein (AtTN10)	Nucleus/Cytoplasm	Plant immunity	Nandety et al., 2013
At1g74050	60S ribosomal protein L6-type (AtRPL6C)	Cytoplasm	Protein synthesis	Carroll, 2013
At1g78630	50S ribosomal protein L13-type	Cytoplasm	Protein synthesis	Carroll, 2013
At2g09990	40S ribosomal protein S16-type (AtRPL16A)	Cytoplasm	Protein synthesis	Carroll, 2013
At2g18710	SECY homolog 1 (AtSCY1)	Chloroplast	Translocation of cytoplasmic proteins into plastid	Skalitzky et al., 2011
At2g20530	Type-II prohibitin (AtPHB6)	Mitochondria	Prohibitin-Cell division	Meyer et al., 2008
At2g20580	RPN1-like regulatory component of 26S proteasome complex	Nucleus/Cytoplasm	Component of 26S proteasome/Plant immunity	Brukhin et al., 2005; Yao et al., 2012
At2g21390	α-subunit of coatomer (COP) adaptor complex	Golgi apparatus	Vesicle Trafficking	Brandizzi and Barlowe, 2013
At3g07110	Ribosomal protein L19-type (AtRPL13aA)	Cytoplasm	Protein synthesis	Millar and Heazlewood, 2003
At3g10860	Ubiquinone-binding component of cytochrome bc1 complex (AtUCR!-1/AtQCR8-1) UCRQ	Mitochondria	Subunit cytochrome bc1 complex/respiration	Meyer et al., 2008
At3g15030	Transcription factor (AtTCP4)	Nucleus	Transcription	Aggarwal et al., 2010
At3g20000	Translocase of the outer mitochondrial membrane 40 (AtTOM40-1)	Mitochondria	Component of the mitochondrial import apparatus	Lister et al., 2004
At3g27240	Cytochrome C1 family (AtCyc1-1)	Mitochondria	Component of the cytochrome C1 subunit	Murcha et al., 2014
At3g29320	Plastidial α-glucan phosphorylase (AtPHS1)	Chloroplast	Abiotic stress	Zeeman et al., 2004
At3g44110	DnaJ homolog 3 (J3)	Plasma membrane	Molecular chaperone	Yang et al., 2010
At3g46740	Translocon at the outer envelope membrane of chloroplasts, 75 kD (AtToc75-III)	Chloroplast	Protein translocation	Baldwin et al., 2005
At3g51820	Chlorophyll synthase (AtChlG)	Chloroplast	Chlorophyll synthase/Chlorophyll biosynthesis	Kim et al., 2013
At3g54110	Mitochondrial uncoupling protein (AtPUMP1/AtUCP1)	Mitochondria	Mitochondrial transport	Monne et al., 2018
At4g13760	Putative polygalacturonase	Extracellular space	Cell wall remodeling	Duan et al., 2016
At4g24190	Sheperd (SHD)	Endoplasmic reticulum	Molecular chaperone	Ishiguro et al., 2002
At4g25030	Arabidopsis thaliana nonhost resistance 2A (AtNHR2A)	Chloroplast	Plant immunity	Singh et al., 2018
At4g26590	OPT-type transporter (AtOPT5)	Plasma membrane	oligopeptide transporter	Lubkowitz, 2011
At4g31990	Aspartate aminotransferase (AtASP5)	Chloroplast	Aspartate biosynthesis	Wilkie et al., 1996
At4g34050	Caffeoyl coenzyme A O-methyltransferase 1 (AtCCoAOMT1)	Cytoplasm	Lignin biosynthesis/Plant immunity	Yang Q. et al., 2017; Liu et al., 2018
At5g01530	Light harvesting complex photosystem II (AtLHCb4.1)	Chloroplast	Light-harvesting chlorophyll a/b binding protein	de Bianchi et al., 2011
At5g12860	Plastidic 2-oxoglutarate/malate translocator (AtDiT1/AtpOMT1)	Chloroplast	Oxaloacetate/malate transporter	Kinoshita et al., 2011
At5g16440	Isopentenyl diphosphate isomerase 1 (AtIDI1)	Chloroplasts	Isopentenyl diphosphate isomerase	Guirimand et al., 2012
At5g19760	Mitochondrial adenine nucleotide transporter (AtDTC)	Mitochondria	Mitochondrial dicarboxylate/Tricarboxylate carrier	Millar and Heazlewood, 2003
At5g33320	Phosphoenolpyruvate/phosphate translocator (AtPPT1/AtCUE1)	Chloroplasts	Plastid metabolism	Flugge et al., 2011
At5g35530	40S ribosomal protein S3 type (AtRPS3C)	Cytoplasm	Protein synthesis	Carroll, 2013
At5g41670	6-phosphogluconate dehydrogenase (AtPGD3)	Cytoplasm, Chloroplast	Putative 6-phospho-gluconate dehydrogenase	Holscher et al., 2016
At5g62790	1-Deoxy-D-xylulose 5-phosphate reductoisomerase (DXR)	Chloroplasts	Isoprenoid biosynthesis	Carretero-Paulet et al., 2002
At5g64940	ABC1-type atypical kinase (AtABC1K8/AtOSA1)	Chloroplast	Prenylquinone synthesis	Manara et al., 2015

**TABLE 3:** List of proteins interacting with AtNHR2A and AtNHR2B with known subcellular localization and function, ordered by gene ID.

**Gene ID**	**Description**	**Subcellular location**	**Function**	**References**
At1g54010	GDSL-like Lipase/Acylhydrolase superfamily protein (AtGLL23)	Endoplasmic Reticulum	Glucosinolate metabolism	Jancowski et al., 2014
At2g20260	Subunit PsaE of photosystem I complex (AtPsaE2)	Chloroplast	Photosynthesis	Ihnatowicz et al., 2007
At2g44530	Phosphoribosyl diphosphate synthetase (AtPRS5)	Chloroplast	Pyrimidine photosynthesis	Howles et al., 2006
At3g08030	Cell wall associated protein	Extracellular space	Plant immunity	Ndimba et al., 2003
At3g08510	Putative phosphatidylinositol-specific phospholipase C (AtPLC2)	Plasma membrane	Auxin biosynthesis and signaling	Otterhag et al., 2001; Li et al., 2015
At3g45140	Putative 13-lipoxygenase (AtLOX2)	Chloroplast	Plant immunity	Bell et al., 1995; Zoeller et al., 2012
At4g13940	S-adenosyl-L-homocysteine hydrolase (AtSAHH1)	Nucleus	Methionine biosynthesis	Lee et al., 2012
At5g00570	Chlorophyll a-b binding protein	Chloroplast	Photosynthesis	Green et al., 1991
At5g55180	Glycosyl hydrolase family 17 protein	Extracellular space	Plant immunity	Zhang et al., 2007

Regarding the subcellular localization of the interactors, we found that the proteins interacting with AtNHR2A and AtNHR2B localized to multiple subcellular compartments. However, the number and specific proteins in each compartment varies when comparing between AtNHR2A- and AtNHR2B-interactors. Among the AtNHR2A interactors, the subcellular compartment containing the largest number of proteins was the cytoplasm followed by the extracellular space (Table 1), while the subcellular compartments containing the largest number of AtNHR2B-interacting proteins were chloroplasts followed by cytoplasm (Table 2). Among the proteins that interacted with both AtNHR2A and AtNHR2B, the most common localization was to chloroplasts followed by extracellular space (Table 3). It was interesting to note that several of the AtNHR2A and AtNHR2B interactors have more than one localization, highlighting that several biological processes require interorganellar movement of proteins.

With regard to functional categories, the most common of them for AtNHR2A interactors was plant immunity and included 15 proteins with demonstrated function in pathogen recognition, regulation of immune responses, signaling, vesicle trafficking, secretion of defense secondary metabolites, and cell wall remodeling. The second most common category for AtNHR2A interactors included 10 proteins participating in enzymatic reactions during primary metabolism, such as synthesis of essential carbohydrates, lipids, or proteins, and occurring in cytoplasm, mitochondria, or chloroplasts. Other categories with significant number of proteins among the AtNHR2A interactors included those associated with RNA-mediated processes, such as transcription, splicing, and RNA stability, as well as those related to protein synthesis, the latter being largely represented by ribosomal proteins. Other categories with fewer proteins included protein folding and turnover, secondary metabolism, hormone-related processes, and abiotic stress (Table 1).

The most common functional category for AtNHR2B interactors was primary metabolism and included 19 proteins with enzymatic activities, as well as structural function, found to be essential in processes occurring in mitochondria and chloroplasts. The second most common category for AtNHR2B interactors was protein synthesis represented by 8 ribosomal proteins (Table 2). In contrast to the abundance of AtNHR2A interactors representing plant immunity, there are only 5 AtNHR2B interactors for the same category which included regulatory proteins, in addition to proteins functioning in pathogen recognition and, significantly, AtNHR2A (Table 2). Other categories represented among the AtNHR2B interactors, but with fewer proteins included vesicle trafficking, cell wall biosynthesis, protein folding and turnover, secondary metabolism, hormone-related processes, and abiotic stress. Two additional categories present among the AtNHR2B interactors but absent among the AtNHR2A interactors included one protein associated with the cytoskeleton and another protein associated with cell division (Table 2).

We found that the 20 proteins that interacted with both AtNHR2A and AtNHR2B can be grouped into the same functional categories as those assigned for proteins interacting with either AtNHR2A or AtNHR2B, those categories being primary metabolism and plant immunity. Other functional categories with fewer proteins were secondary metabolism and hormone-related processes (Table 3).

Although we were able to reliably assign subcellular localization and function to 62% of the proteins interacting with AtNHR2A, 66% of the proteins interacting with AtNHR2B, and 45% of the proteins interacting with both AtNHR2A and AtNHR2B, there were still many interacting proteins that were classified as unknown, either because they are truly unknown, or because published research on those proteins did not provide compelling evidence for their subcellular localization and/or function.

Altogether, the differences between the AtNHR2A and AtNHR2B interactomes demonstrates that AtNHR2A and AtNHR2B have distinct functions. However, the finding that they interact with common proteins, and that the non-common proteins participate in common functional processes, indicate that AtNHR2A and AtNHR2B act together as previously proposed (Singh et al., 2018).

### Validation of Selected Interaction Pairs by Co-immunoprecipitation

**FIGURE 2:**
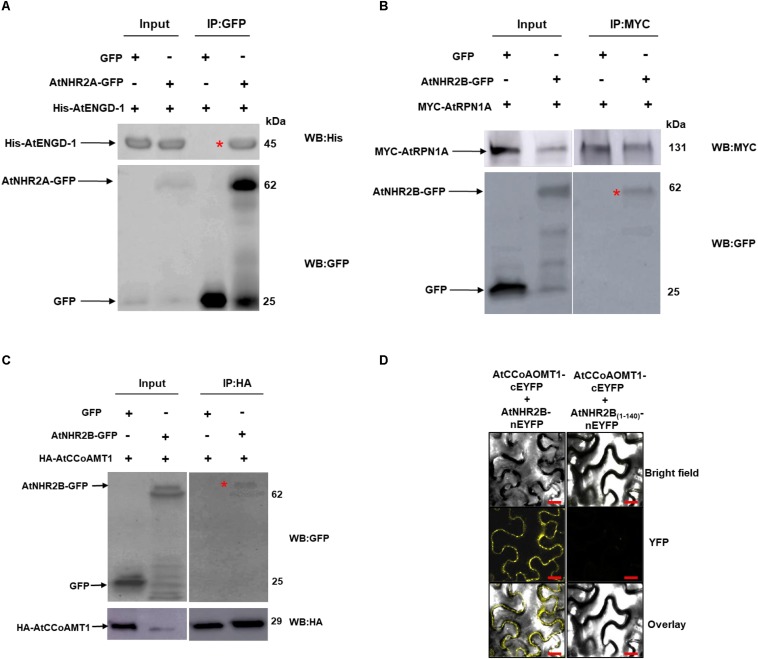
Validation of selected AtNHR2A and AtNHR2B interactions. Protein extracts of *N. benthamiana* transiently expressing AtNHR2A-GFP were mixed with His-AtENGD-1 purified from *E. coli* (Rosetta) and subjected to immunoprecipitation with anti-GFP antibodies. Co-immunoprecipitation of His-AtENGD-1 (red asterisk) was detected by Western blot using anti-His antibodies **(A)**. AtNHR2B-GFP and Myc-AtRPN1A pair and AtNHR2B-GFP and HA-AtCCoAMT1 pair were transiently co-expressed in *N. benthamiana* and immunoprecipitated with anti-Myc and anti-HA antibodies, respectively. Co-immunoprecipitation of AtNHR2B-GFP in each experiment (red asterisks) were detected by Western blot using anti-GFP antibodies **(B,C)**. Expected protein sizes are shown by arrows. AtNHR2B, or its non-functional version AtNHR2B_(1__–__140)_ fused to the N-terminal fragment of EYFP were co-expressed in *N. benthamiana* with AtCCoAOMT1 fused to the C- terminal fragment of EYFP. The reconstitution of the EYFP signal was evaluated via laser scanning confocal microscopy at 3 days after infiltration **(D)**. Images were taken using excitation wavelength of 514 nm and an emission wavelength of 500 to 530 nm. Bar = 10 μm.

To further confirm the accuracy of the IP-MS method, and to gain insight into the function of AtNHR2A and AtNHR2B in plant immunity, we chose to validate the interaction between AtNHR2A or AtNHR2B with proteins known to function in plant innate immunity such as AtENGD-1, AtRPN1A, and AtCCoAMT1 (Yao et al., 2012; Abdeeva et al., 2018; Xie et al., 2018). AtENGD-1 was fused to the 6× histidine (6× His) tag and expressed and purified from *E. coli*. Purified His- AtENGD-1 was added to protein extracts obtained from *N. benthamiana* plants expressing AtNHR2A-GFP. Immunoprecipitation of AtNHR2A-GFP with GFP antibodies, co-immunoprecipitated His- AtENGD-1, as shown by Western blot analysis using anti-His antibodies. Anti-GFP antibodies also immunoprecipitated GFP but did not co-immunoprecipitated His- AtENGD-1 (Figure 2A and Supplementary Figure S3).

The interaction between AtNHR2B-GFP and AtRPN1A, and that between AtNHR2B-GFP with AtCCoAOMT1 was validated by reciprocal co-IP, wherein *AtRPN1A* and *AtCCoAOMT1* were cloned as fusions to the MYC and HA tags, respectively, and used to pull down AtNHR2B-GFP. Immunoprecipitation of MYC-AtRPN1A with anti-MYC antibodies co-immunoprecipitated AtNHR2B-GFP but not GFP alone (Figure 2B and Supplementary Figure S4). Immunoprecipitation of HA-AtCCoAOMT1 with anti-HA antibodies also co-immunoprecipitated AtNHR2B-GFP but not GFP alone (Figure 2C and Supplementary Figure S5).

### Validation of Protein–Protein Interaction by Bimolecular Fluorescence Complementation

We also used bimolecular fluorescence complementation (BiFC) in *N. benthamiana* to confirm the interaction between AtNHR2B-GFP and AtCCoAOMT1 *in planta.* For that purpose, full length AtNHR2B was fused to the N-terminal half of the enhanced yellow fluorescent protein (*nEYFP*), while *AtCCoAOMT1* was fused to the C-terminal half of *EYFP* (*cEYFP*). Following the published guidelines for BIFC (Bock and Kudla, 2016), a truncated version of AtNHR2B was used as control containing only the first 140 amino acids (AtNHR2B_1__–__140_) as this truncation abolishes normal localization of AtNHR2B-GFP (Singh and Rojas, 2018). AtNHR2B_1__–__140_ was fused to *nEYFP*, and AtNHR2B-nEYFP or AtNHR2B_(__1__–__140__)_-nEYFP were transiently co-expressed with AtCCoAOMT1-cEYFP in *N. benthamiana* to examine the reconstitution of the EYFP fluorescence upon interaction between the two proteins. Co-expression of full length AtNHR2B-nEYFP with AtCCoAOMT1-cEYFP generated a yellow fluorescence signal indicating the reconstitution of the EYFP by the interaction between full length AtNHR2B and AtCCoAOMT1 *in planta*. Moreover, this BiFC data clearly demonstrated that the interaction between full length AtNHR2B and AtCCoAOMT1 occurs in the cytoplasm and punctae bodies where AtNHR2B has been consistently found (Singh et al., 2018). In contrast, co-expression of AtNHR2B_(__1__–__140__)_ –nEYFP with AtCCoAOMT1-cEYFP did not show yellow fluorescence (Figure 2D) indicating that the truncation of AtNHR2B interferes with the interaction.

### AtNHR2A and AtNHR2B Belong to the Same Network

**FIGURE 3:**
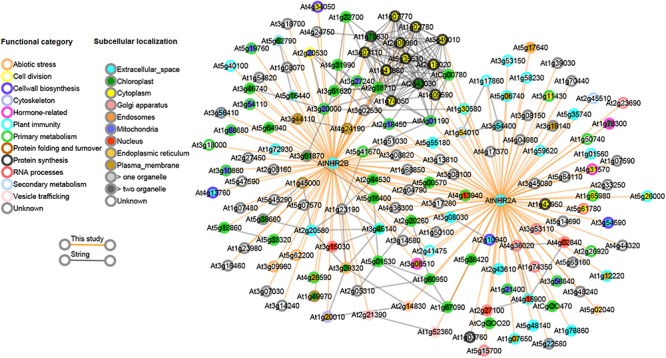
Combined interactome network for AtNHR2A and AtNHR2B. The AtNHR2A-AtNHR2B interactome was combined with predicted gene functional associations in the STRING database and visualized using Cytoscape v 3.3.0. Circles represent genes color-coded for functional category and subcellular localization based on literature searches. Orange lines show the interactions between each gene and AtNHR2A and/or AtNHR2B using IP-MS. Gray lines show predicted functional interactions using STRING network.

Our finding that AtNHR2A and AtNHR2B share a common set of interacting proteins, together with our accumulated genetic evidence (Singh et al., 2018), implies that AtNHR2A and AtNHR2B belong to the same protein network. Thus, we investigated if the observed links in our protein interaction data were also preserved in other protein networks. We reasoned that since proteins in a subnetwork are more likely to be functionally related, the interactions we observed in our IP-MS dataset should also be predictable from other data sources. To test this, we obtained high-confidence Arabidopsis protein network data from the STRING database (Szklarczyk et al., 2019). The protein links represented in the STRING database were computationally predicted and scored by combining probabilities depicting functional-relatedness from seven different evidence channels in Arabidopsis. Overlapping this predicted network with our data revealed that several of the AtNHR2A- and AtNHR2B- interactors that were experimentally identified in this study were also preserved in the STRING network data (Figure 3). Specifically, out of 155 proteins in our interactome, ∼40% are also connected to each other in the STRING network, indicating that these are genuine interactions. Remarkably, a large fraction of the proteins that overlapped with the predicted network are the proteins that are common for AtNHR2A and AtNHR2B with a large fraction of these proteins being involved in protein synthesis. The network also shows a preferred subcellular localization to chloroplasts among the AtNHR2B interactors.

## Discussion

In this study, we unraveled the interactomes of AtNHR2A and AtNHR2B to gain further insight into their detailed function so as to integrate this knowledge with our previous live-cell imaging data and with the biological phenotypes for *AtNHR2A* and *AtNHR2B*. Our *in vivo* IP-MS used conditions that directly reflect *AtNHR2A* and *AtNHR2B* expression and their corresponding endogenous protein abundance while eliminating false positives due to non-specific interactions with the epitope tag (GFP). We found that proteins interacting with AtNHR2A and AtNHR2B are localized to diverse subcellular compartments with a preferred localization to cytoplasm and chloroplasts. These findings are not surprising considering our previous live-cell imaging data indicated that AtNHR2A and AtNHR2B localize to these compartments (Singh et al., 2018). The localization of AtNHR2A and AtNHR2B to chloroplasts is important in the context of plant immunity as this organelle provides key defense signaling molecules, such as hormones and reactive oxygen species (Serrano et al., 2016). However, none of the chloroplastic-localized proteins that interacted with AtNHR2A or AtNHR2B are known to play a role in chloroplast-mediated immune responses. Rather, all the AtNHR2A and AtNHR2B interactors that localized to chloroplasts are implicated in primary metabolism, functioning either as enzymes or as structural components. Thus, the localization of AtNHR2A and AtNHR2B to chloroplasts is still mysterious. Nevertheless, because AtNHR2B interacted with more chloroplastic proteins than AtNHR2A, it is likely that AtNHR2B plays a more prominent function than AtNHR2A in chloroplasts. Interestingly, chloroplasts were also the preferred localization for proteins that interacted with both AtNHR2A and AtNHR2B, and those common interactors can be seen as bridges integrating both independent interactomes.

One curious finding was the abundance of AtNHR2A- and AtNHR2B- interacting proteins localized to the extracellular space, particularly among the AtNHR2A interactors. Several of those extracellularly localized proteins included proteins functioning in plant immunity either as antimicrobials, such as chitinase (Kumar et al., 2018), or participating in cell wall biosynthesis or remodeling, such as pectin lyase and endo-1,3-beta-glucosidase (Cao, 2012; Houston et al., 2016). AtNHR2B also interacted with a putative polygalacturonase (Duan et al., 2016). Other AtNHR2A interactors localized to the extracellular space and functioning in cell wall biosynthesis included the extensin AtEXT2 (Liepman et al., 2010), and a proline-rich glycoprotein (Durufle et al., 2017). However, we did not find experimental evidence that these two proteins participate in plant immunity.

In addition to cell wall-related proteins localized to the extracellular space, AtNHR2A and AtNHR2B interacted with cytoplasmic proteins that also contribute to cell wall remodeling. AtNHR2A interacted with the thioglucoside glucohydrolase (AtTTG1), a myrosinase that functions in the hydrolysis of glucosinolates (Grubb and Abel, 2006) and the biosynthesis of callose (Clay et al., 2009). AtNHR2B interacted with Caffeoyl coenzyme A O-methyltransferase 1 (AtCCoAOMT1), an enzyme that participates in the biosynthesis of lignin, a component of the secondary plant cell wall (Liu et al., 2018). *AtCCoAOMT1* has recently being implicated in plant immunity since this gene is induced by pathogens, and *Atccoamt1* mutants are more susceptible to infections by *P. syringae* pv. tomato and *Hyaloperonospora arabidopsidis* (Yang Q. et al., 2017). The commonalities between AtNHR2B and AtCCoAOMT1 prompted us to validate this interaction *in vivo* by two independent methods. One method included transient co-expression of AtNHR2B-GFP and HA-AtCCoAOMT1 in *N. benthamiana* followed by co-immunoprecipitation, but using HA-AtCCoAOMT1 as bait to co-immunoprecipitate AtNHR2B-GFP in contrast to the original screen that used AtNHR2B-GFP as bait. We also validated this interaction *in vivo* by BiFC and demonstrated that AtNHR2B and AtCCoAOMT1 interacted in the cytoplasm and punctate bodies, which agrees with the expected localization of AtNHR2B. The validation of this interaction will pave the way to continue exploring how AtNHR2B and AtCCoAOMT1 work together during plant immunity.

Cell wall biosynthetic enzymes and antimicrobials are localized to the extracellular space because they are secreted proteins that transition through a secretory pathway involving the endomembrane system. Our findings that AtNHR2A and AtNHR2B interacted with extracellular proteins suggest either that AtNHR2A and/or AtNHR2B are also secreted proteins or that AtNHR2A and AtNHR2B are components of the secretory pathway. Although we do not have evidence yet for the secretion of AtNHR2A or AtNHR2B, our interactome data indicates that both AtNHR2A and AtNHR2B interacted with proteins associated with secretory processes. AtNHR2A interacted with the Prenylated Rab acceptor 1 (AtPRA1.A1) and with AtISTL10, two proteins that participate in membrane trafficking events through distinct mechanisms (Alvim Kamei et al., 2008; Buono et al., 2016). In addition, both AtNHR2A and AtNHR2B interacted with the coat protein complex I (COPI), a complex of 7 proteins (α, β.β’, γ,δ,ε,ζ) that transports cargo from the Golgi apparatus to the ER (Brandizzi and Barlowe, 2013). Interestingly, AtNHR2A interacted with the β subunit while AtNHR2B interacted with the α subunit, and the interaction between the α and β subunits was also uncovered with the interactome network. These results combined with our previous live-imaging data showing localization of AtNHR2A and AtNHR2B to small punctae resembling bodies of the endomembrane system (Singh et al., 2018) strongly suggests that AtNHR2A and AtNHR2B are involved in endomembrane trafficking related events and supports our previous hypothesis, based on genetic data, that AtNHR2A and AtNHR2B work together (Singh et al., 2018).

We also identified as AtNHR2A and AtNHR2B interactors, other proteins known to participate in plant immunity at multiple levels including pathogen recognition and regulation of immune processes. Regarding pathogen recognition, we found that AtNHR2A interacted with the lectins AtLecRK-S.5 and AtGAL2 (Hwang and Hwang, 2011; Lannoo and Van Damme, 2014) and three resistance genes: AtRPS5 (Qi et al., 2012), an NB-ARC type disease resistant protein (van Ooijen et al., 2008), and BRS650, a LRR-type receptor protein kinase functioning in brassinosteroid signaling during plant immunity (Xu et al., 2014). AtNHR2B also interacted with AtTN10, a type of nucleotide binding-leucine rich repeat (NLR) recognizing pathogen effectors (Nandety et al., 2013).

With respect to the interaction of AtNHR2A and AtNHR2B with proteins known to be important in regulation of plant immunity, we found that AtNHR2A interacted with AtPRL1, a protein that interacts with the plant defense transcription factor AtCDC5 (Palma et al., 2007), with the MAP kinase AtMPK11 (Carrasco et al., 2014), and with AtENGD-1 (Cheung et al., 2010). AtENGD-1 is a GTP-binding protein that participates in plant immunity regulating the production of ROS and interacting with the immunophilin AtCYP19, which is also important for plant defense responses against *P. syringae* (Cheung et al., 2010; Pogorelko et al., 2014; Abdeeva et al., 2018). We successfully validated the interaction between AtNHR2A and AtENGD-1 by semi- *in vivo* co-immunoprecipitation wherein His-AtENGD-1 purified from bacteria was successfully co-immunoprecipitated by AtNHR2A-GFP expressed and purified from *N. benthamiana.* AtNHR2B also interacted with proteins known to play regulatory roles in plant immunity, specifically the ATL-RING-H2-E3 ubiquitin ligase (Liu et al., 2008) and AtRPN1, a component of the 26S proteasome subunit (Brukhin et al., 2005). AtRPN1 is required for resistance against fungal and bacterial pathogens (Yao et al., 2012). We also validated the interaction between AtNHR2B and AtRPN1 by using Myc-AtRPN1 as a bait to co-immunoprecipitate AtNHR2B-GFP. The validations of two of these interactions between AtNHR2A or AtNHR2B and proteins known to participate in plant immunity not only supports the quality of the data obtained by IP-MS, but also provided further insight into the multiple functions of AtNHR2A and AtNHR2B in plant immunity.

Because AtNHR2A and AtNHR2B were initially identified as important components of plant immunity, the main focus of this research was to unravel immune-related processes in which these two proteins participate. Indeed, this study found that AtNHR2A and AtNHR2B interact with a significant number of proteins known to participate in plant immunity, and, remarkably, cover a wide range of processes encompassing pathogen recognition, regulation of immune responses, and deployment of defense molecules with antimicrobial properties or functioning in the strengthening of the plant cell wall. Although careful validation of other interactions is needed, we are confident that this study provided a solid ground to generate testable hypotheses that will enable us to further dissect the complexity of plant immunity. Intriguingly, this study also found several other proteins that do not appear to function in plant immunity, highlighting the dynamic and versatile nature of AtNHR2A and AtNHR2B, possibly working as hubs integrating plant immunity with other biological processes (Vandereyken et al., 2018).

## Data Availability Statement

The datasets generated for this study can be found in the PRIDE archive using accession number PXD015894.

## Author Contributions

RS, JL, and CR designed the study. RS and CR wrote the manuscript. RS and RL performed experiments, collected and analyzed data. CG and AP analyzed data. All authors read and approved the manuscript.

## Conflict of Interest

The authors declare that the research was conducted in the absence of any commercial or financial relationships that could be construed as a potential conflict of interest.

## Footnotes

1 https://www.arabidopsis.org/

2 https://abrc.osu.edu/
