# Supplementary material for: The Arabidopsis Proteins AtNHR2A and AtNHR2B Are Multi-Functional Proteins Integrating Plant Immunity With Other Biological Processes
Source: Front Plant Sci. 2020 Mar 4;11:232. doi: 10.3389/fpls.2020.00232 (PMC7064621; doi:10.3389/fpls.2020.00232)
Supplement: Supplementary file 1 [file Data_Sheet_1.DOCX]

Supplementary Material

**
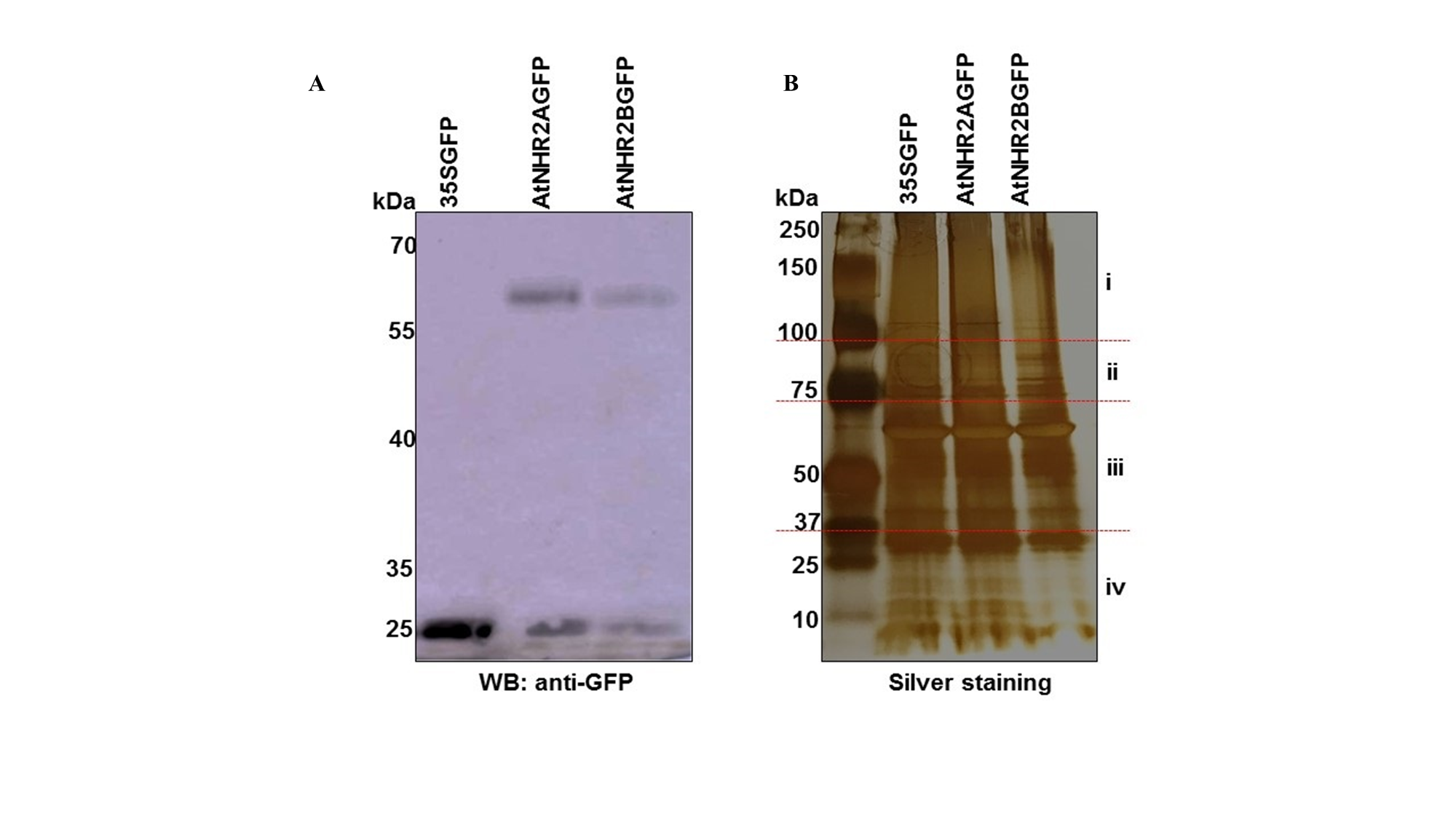
Supplementary Figure 1. Protein expression analysis of transgenic Arabidopsis lines expressing AtNHR2A-GFP and AtNHR2B-GFP and free GFP.** Four-week-old plants were inoculated with *Pseudomonas syringae* pv tabaci (1 X 10^6^  CFU/ml) and harvested at 6 hpi for protein extraction. Extracted proteins were separated on mass spectrometry compatible 4-20% Tris-Glycine gel and transferred to a nitrocellulose membrane for Western blot with anti-GFP-HRP antibodies (A). Total proteins were immunoprecipitated with GFP-Trap A beads and eluted with 2X SDS buffer. The eluted proteins were resolved on 4-20% Tris-Glycine Gel and visualized by silver staining. Red lines show the molecular weight ranges for four fractions (i, ii, iii, iv) that were cut out from the gel for each sample before trypsin digestion (B).


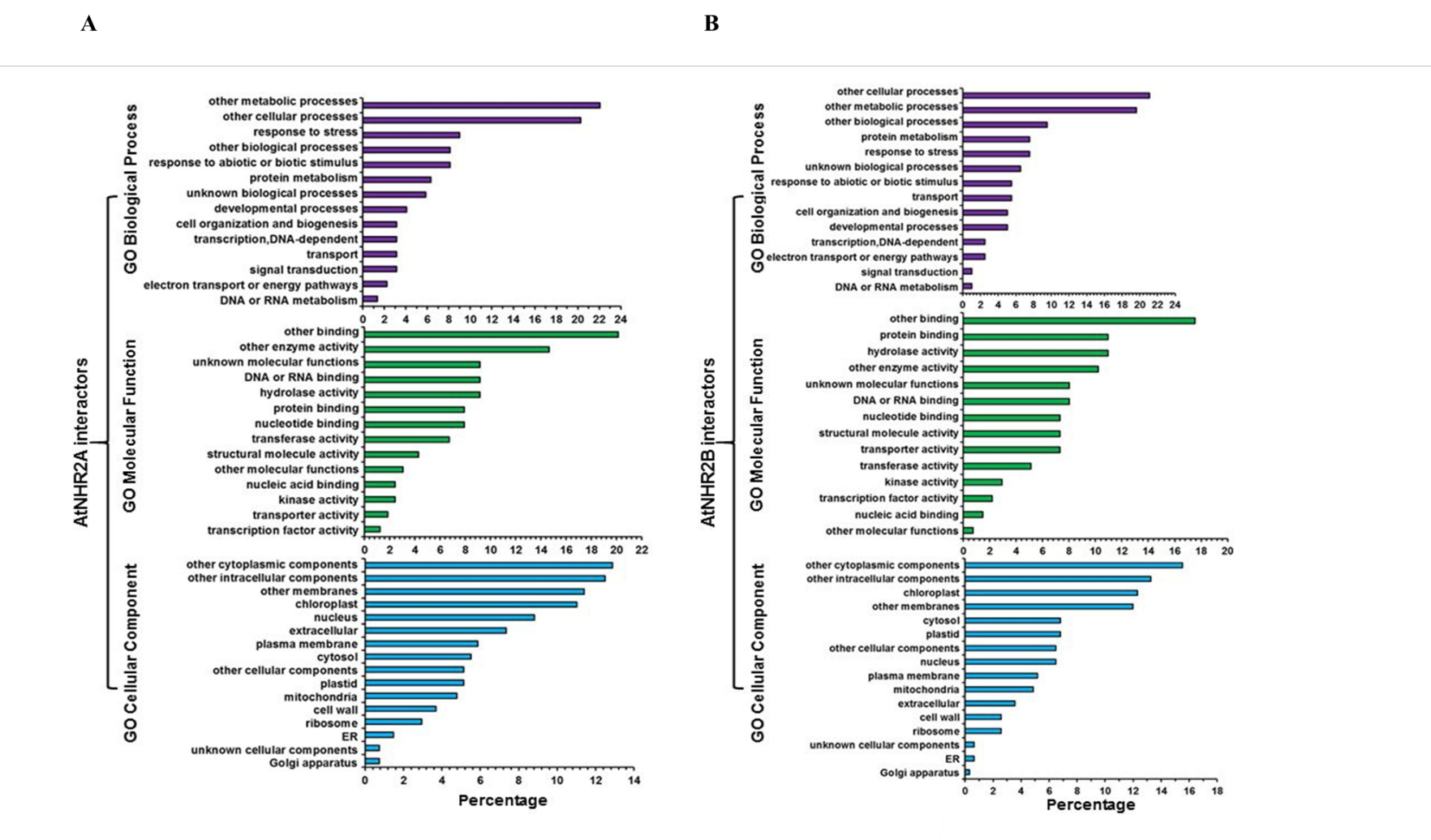


**Supplementary Figure 2. Proteins interacting with AtNHR2A and AtNHR2B are involved in distinct biological processes.** The Gene Ontology (GO) annotation tool was used to classify AtNHR2A-interacting proteins (A) and AtNHR2B-interacting proteins (B) into three GO categories: biological function, molecular function and cellular component.


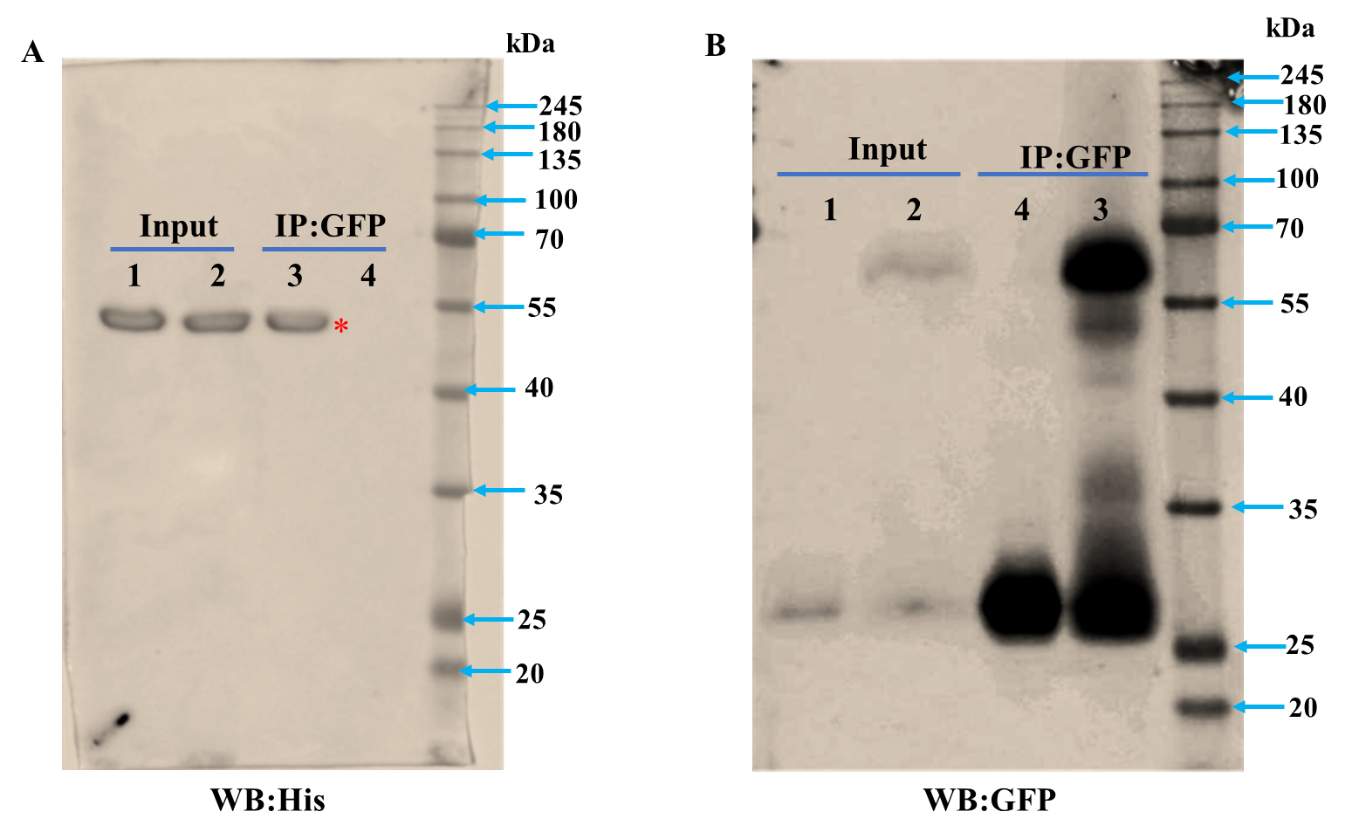


**Supplementary Figure 3. Western blots showing co-immunoprecipitation of AtNHR2B-GFP by His-AtENGD-1 as detected by His antibody.** Input and co-immunoprecipitated samples were run onto an SDS-PAGE gel and transferred to a nitrocellulose membrane for Western blot analysis using anti-His antibodies to detect His-AtENGD-1 (A) and anti-GFP antibodies to detect AtNHR2A-GFP and GFP (B). Lane 1: GFP + His-AtENGD-1; Lane 2: AtNHR2A-GFP + His-AtENGD-1; Lane 3: AtNHR2A-GFP + His-AtENGD-1; Lane 4: GFP + His-AtENGD-1.


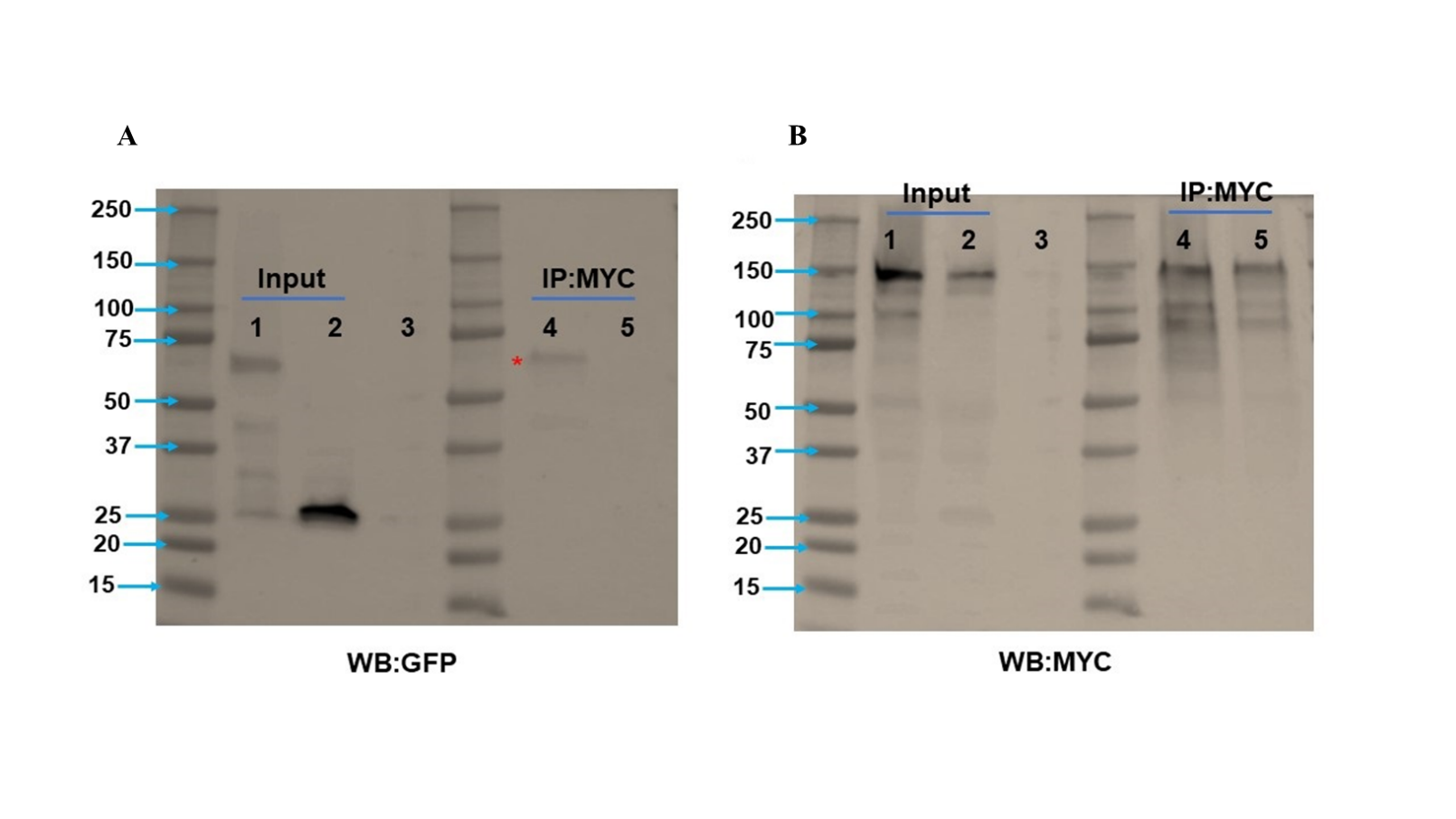


**Supplementary Figure 4. Western blots showing co-immunoprecipitation of AtNHR2B-GFP by MYC-AtRPN1A as detected by GFP antibody.** Input and co-immunoprecipitated samples were run onto an SDS-PAGE gel and transferred to a nitrocellulose membrane for Western blot analysis using anti- GFP antibodies to detect AtNHR2B-GFP and GFP (A) and anti-MYC antibodies to detect MYC-AtRPN1A (B). Lane 1: AtNHR2B-GFP + MYC-AtRPN1A; Lane 2: GFP + MYC-AtRPN1A; Lane 3: empty; Lane 4: AtNHR2B-GFP + MYC-AtRPN1A; Lane 5: GFP + MYC-AtRPN1A.


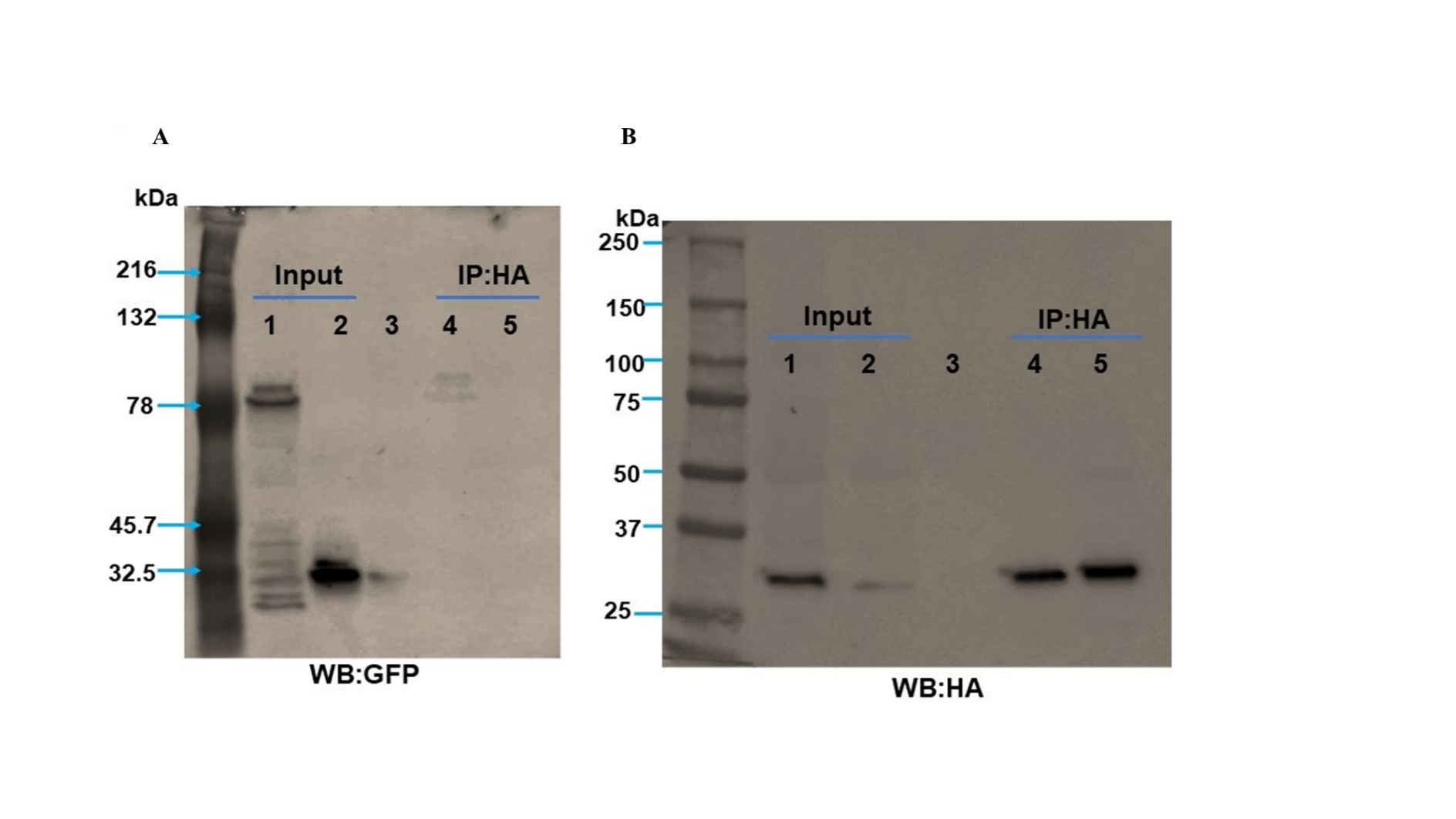


**Supplementary Figure 5. Western blots showing co-immunoprecipitation of AtNHR2B-GFP by HA-AtCCoAMT1 as detected by GFP antibody**. Input and co-immunoprecipitated samples were run onto an SDS-PAGE gel and transferred to a nitrocellulose membrane for Western blot analysis using anti- GFP antibodies to detect AtNHR2B-GFP and GFP (A) and anti-HA antibodies to detect HA- AtCcoAMT1(B). Lane 1: AtNHR2B-GFP + HA-AtCCoAMT1; Lane 2: GFP + HA-AtCCoAMT1; Lane 3: empty; Lane 4: AtNHR2B-GFP + HA-AtCCoAMT1; Lane 5: GFP + HA-AtCCoAMT1.
